# Application of chromosomal microarray analysis for prenatal diagnosis in 315 ultrasonically abnormal fetuses

**DOI:** 10.3389/fgene.2026.1744468

**Published:** 2026-03-25

**Authors:** Zhiyuan Zheng, Heming Wu, Lingna She, Dandan Luo, Lifang Lin, Wei Guo, Liubing Lan

**Affiliations:** 1 Department of Prenatal Diagnostic Center, Meizhou People’s Hospital, Meizhou Academy of Medical Sciences, Meizhou, China; 2 Meizhou Municipal Engineering and Technology Research Center for Molecular Diagnostics of Major Genetic Disorders, Meizhou People’s Hospital, Meizhou Academy of Medical Sciences, Meizhou, China; 3 Department of Ultrasound, Meizhou People’s Hospital, Meizhou Academy of Medical Sciences, Meizhou, China

**Keywords:** chromosomal microarray analysis, copy number variant (CNV), karyotype, prenatal diagnosis, ultrasonic abnormality

## Abstract

The purpose of this study was to assess the application value of chromosome microarray analysis (CMA) for prenatal diagnosis of fetuses with ultrasonic abnormalities. A retrospective study was conducted on 315 fetuses with ultrasonic abnormalities without aneuploidies who received prenatal diagnosis at Meizhou People’s Hospital, from October 2022 to December 2023. Fetal specimens obtained by ultrasound guided puncture were detected by CMA analysis with Affymetrix CytoScan 750K array. The detection rate of chromosomal abnormalities in different ultrasonic abnormalities was analyzed. Among the 315 fetuses, 16 (5.08%) were detected with pathogenic/likely pathogenic copy number variants (P/LP CNVs). Three (5.88%) among 51 cases with ultrasound structural abnormalities in multiple organ systems were detected with P/LP CNVs, 5 (6.02%) among 83 cases with a single structural anomaly were detected with P/LP CNVs, and 8 (4.42%) among 181 cases with ultrasonographic soft markers were detected with P/LP CNVs. Compared with conventional karyotyping analysis, CMA can improve the detection of fetal chromosomal abnormalities and provide an effective diagnostic tool for prenatal diagnosis. Chromosomal microarray analysis; Ultrasonic abnormality; Karyotype; Prenatal diagnosis.

## Introduction

Birth defects, also known as congenital abnormalities, refer to structural, functional, or metabolic abnormalities that occur before birth (Khokha and Liu, 2020; Banu, 2024). The incidence of birth defects in China is about 5.6%, and the prevention and control situation of birth defects is not optimistic (Zhang et al., 2023). Prenatal diagnosis is a specialized medical procedure that involves the application of multiple technical approaches—including imaging techniques, biochemical assays, cytogenetic analysis, and molecular biological testing—to assess fetal growth and development, and identify congenital malformations or hereditary disorders prior to birth (Luo et al., 2023). The core objective of prenatal diagnosis is to prevent the birth of infants with congenital defects and improve the overall population quality (Duyzend and Cacheiro, 2024). With the continuous progress of medical technology, a variety of detection methods have been widely used in prenatal diagnosis, in order to detect congenital and genetic diseases of the fetuses as early as possible, and provide scientific basis for subsequent intervention measures (Bedei et al., 2021; Oyelese et al., 2025).

Ultrasonic detection has become one of the key means of prenatal diagnosis due to its advantages of simplicity, non-invasion and high repeatability (Ramirez Zegarra and Ghi, 2023). During pregnancy, ultrasound can visually observe the morphological structure of fetuses, and effectively screen out most obvious fetal structural abnormalities, such as congenital heart disease (CHD) (Yang et al., 2025), neural tube defects (Didier and Martin-Saavedra, 2020), and limb malformations (Thakur et al., 2023). For such serious fatal and disabling structural abnormalities, pregnant women can consider terminating pregnancy according to the diagnosis results and under the premise of being fully informed, thus greatly reducing the birth rate of children with birth defects. However, ultrasonic detection is not perfect and has some limitations. In practical applications, it is often difficult to make a clear diagnosis of small structural abnormalities such as mild ventricular dilatation, ventricular bright spot, renal pelvis dilatation, and ultrasonographic soft markers abnormality solely through ultrasound examinations, which brings great challenge to clinical decision-making (Moradi et al., 2024). The traditional chromosomal karyotype analysis technique, regarded as the gold standard of prenatal fetal chromosome examination, has played an important role in detecting large structural rearrangement of chromosomes and aneuploidy (Kamath et al., 2022). However, this technology can easily cause missed diagnosis for chromosome microdeletions and microduplications, and it is difficult to meet the growing demand for accurate prenatal diagnosis (Van den Veyver et al., 2022).

Chromosomal microarray analysis (CMA) is a chip based technology that can simultaneously detect copy number variants (CNVs) by fixing a large number of known DNA probes to the surface of the chip (Kim et al., 2024; Kang et al., 2024). CMA can effectively detect chromosome microdeletions and microduplications that cannot be detected by chromosomal karyotype analysis, and greatly improve the detection resolution of chromosome abnormalities (Walton et al., 2023). With the feature of whole genome coverage, CMA technology can detect a large number of chromosome loci in a single experiment, providing a powerful tool for the study of chromosome microstructure changes, and CMA has become a first-line detection technology in the field of prenatal diagnosis (Lan et al., 2024; Prenatal et al., 2023). Exploring the application of CMA in the prenatal diagnosis of fetuses with abnormal ultrasonic findings is of great significance for improving the accuracy of prenatal diagnosis, optimizing clinical decision-making, and reducing the birth rate of infants with congenital defects.

## Materials and methods

### Study cohort and data collection

This study conducted a retrospective analysis of 315 fetuses whose ultrasound examination results were abnormal and underwent CMA analysis at the Prenatal Diagnosis Center of Meizhou People’s Hospital from October 2022 to December 2023. These fetuses are consecutive cases within this period. This study was approved by the Medical Ethics Committee of Meizhou People’s Hospital. The mean age of the pregnant women was 29.5 years, and the mean gestational age was 22 weeks and 5 days. All 315 fetuses were found to have no obvious abnormality by conventional karyotype analysis, and the above cases were analyzed by CMA. Genetic detection of fetal specimens (villi, amniotic fluid, and umbilical cord blood) obtained by ultrasound-guided puncture was carried out using G-banding karyotype analysis and CMA (Affymetrix Cytoscan 750K).

In this study, fetal ultrasound abnormalities included: (1)fetal ultrasound structural abnormalities (Buijtendijk et al., 2024): ultrasound indicated the abnormality of fetal anatomical structure, including cardiovascular system, urinary system, thoracic, cephalic facial, nervous system, digestive system, skeletal system, abdominal wall, and other malformations; (2) ultrasound soft markers, including nuchal translucency (NT) thickening, ventricular bright spot, nasal bone dysplasia, echogenic bowel, mild ventricular dilatation, posterior fossa cistern widening, choroid plexus cyst, pyelic separation, short femur, and single umbilical artery (Kim et al., 2024; Liu et al., 2025). Fetal single system ultrasound structural abnormality refers to the condition where only a single organ of the fetus is found to have structural malformation via prenatal ultrasound examination, with no abnormal indicators detected in other organs or systems. Fetal multi-system ultrasound structural abnormalities refers to a type of complex ultrasonic anomaly in which structural malformations are identified in two or more different organs or systems of the fetus via prenatal ultrasound examination.

Inclusion criteria: (1) structural abnormalities on ultrasound, including single-system or multi-system structural abnormalities, with or without non-structural abnormalities such as intrauterine growth retardation and polyhydramnios/oligohydramnios (Qin et al., 2023; Qi et al., 2024); and (2) gestational age between 12 and 34 weeks. Exclusion criteria: (1) fatal ultrasound structural abnormalities, including anencephaly, severe encephalocele, severe open spina bifida, severe chest or abdominal wall defect, internal organ inversion, single-ventricle heart, fatal achondroplasia (Bijok et al., 2023; Zou et al., 2020; Freud et al., 2022); and (2) there were contraindications to amniocentesis (Zemet et al., 2025).

### CMA testing

Fetal specimens (villi, amniotic fluid, and umbilical cord blood) obtained by ultrasound-guided puncture. Genomic DNA was extracted using QIAamp DNA Blood Mini Kit (Qiagen, Germany). Nano Drop2000 (Thermo Fisher scientific Inc, USA) was used to measure DNA concentration to ensure DNA concentration greater than 50 ng/uL with A260/A280 in the range of 1.8–2.0. Prior to CMA testing, amniotic fluid and chorionic villus samples were screened for maternal cell contamination by quantitative fluorescent PCR (QF-PCR). This approach effectively reduces the risk of misdiagnosis caused by maternal cell contamination and ensures the accuracy and reliability of prenatal diagnostic results.

Genomic DNA was performed CMA analysis using Affymetrix Cytoscan 750K chip. Molecular karyotype analysis was performed using the Chromosome Analysis Suite (ChAS) v4.1 (Thermo Fisher Scientific). The detected CNVs were analyzed and interpreted in combination with public databases commonly used internationally, such as the University of California Santa Cruz Database (UCSC) (https://genome.ucsc.edu), Database of Genomic Variation and Phenotype in Humans using Ensembl Resources (DECIPHER) (http://decipher.sanger.ac.uk), Clinical Genome Resource (ClinGen) (https://www.clinicalgenome.org/), Database of Genomic Variants (DGV) (http://dgv.tcag.ca/dgv/app/homr), and Online Mendelian Inheritance Database in Man (OMIM) (https://www.omim.org). According to the American College of Medical Genetics and Genomics (ACMG) guidelines, the clinical significance of CNVs is divided into 5 grades: pathogenic (P) CNV, likely pathogenic (LP) CNV, variants of uncertain significance (VUS) CNV, likely benign (LB) CNV, and benign (B) CNV(Riggs et al., 2020; Brandt et al., 2020).

### Data analysis

In this study, descriptive statistical methods were used to collate and analyze the data, aiming to clearly show the detection rate of CMA in the prenatal diagnosis of fetal ultrasound abnormalities.

## Results

### Results of CNVs by CMA detection for fetuses with different ultrasound abnormalities

Among 315 fetuses with abnormal ultrasound findings, there were 51 cases of multi-system structural abnormalities, among which the most common was nervous system structural abnormalities/cardiovascular structural abnormalities combined with skeletal system structural abnormalities (51 cases). CMA showed 3 cases of P/LP CNVs, 3 cases of VUS, and 45 cases of B/LB CNVs, with an abnormal rate of 5.88%. There were 83 cases of single system ultrasound structural abnormalities, and the most common structural abnormalities were nervous system abnormalities (25 cases). CMA detected 5 cases of P/LP CNVs, 3 cases of VUS, and 75 cases of B/LB CNVs, with an abnormal rate of 6.02%. There were 181 cases of abnormal ultrasound soft markers, including increased nuchal translucency (NT) thickening (40 cases) and mild ventricular dilatation (38 cases). CMA detected 8 cases of P/LP CNVs, 9 cases of VUS and 164 cases of B/LB CNVs, with an abnormal rate of 4.42% (Table 1).

**TABLE 1:** The results of CMA detetion in various prenatal diagnostic indications.

Indications	Number of cases	The results of CMA detetion (n,%)		
		P/LP CNVs (%)	VUS (%)	B/LB CNVs (%)
Multiple system ultrasound abnormalities	51	3 (5.88)	3 (5.88)	45 (88.23)
Single system ultrasound abnormalities	83	5 (6.02)	3 (3.61)	75 (90.36)
Abnormal ultrasound soft markers	181	8 (4.42)	9 (4.97)	164 (90.60)
Total	315	16 (5.08)	15 (4.76)	284 (90.16)

CMA, chromosomal microarray analysis; P/LP CNVs, pathogenic/likely pathogenic copy number variants; VUS, variants of uncertain significance; B/LB CNVs, benign/likely benign copy number variants.

### Detection results and outcomes of 16 fetuses with pathogenic CNVs detected

P/LP CNVs were detected in 16 of 315 amniotic fluid samples, with an abnormal rate of 5.08%. In 6 cases, the peripheral blood of the couple was collected for CMA detection. Two cases were *de novo* and four cases were inherited (Table 2).

**TABLE 2:** Detection results and outcomes of 16 fetuses with pathogenic CNVs detected.

Case	Ultrasound findings	CMA result	Fragment size	De novo/Inherited	Database query results	Follow-up outcome
1	Tricuspid regurgitation	arr7q11.23 (72,701,099-74154209)×1	1.45 Mb	Unknown	Pathogenicity Williams - beuren syndrome	Termination
2	Widened the right ventricle	arrXp22.31 (6449837-8143509)×1	1.69 Mb	De novo	Pathogenicity X-linked ichthyosis	No obvious abnormalities after delivery
3	Tricuspid regurgitation	arrXp21.1 (31750507-31889320)×1	139 Kb	Unknown	Pathogenicity Duchenne muscular dystrophy (DMD)/Becker muscular dystrophy (BMD)	Termination
4	Developmental retardation	arr22q11.21 (18631365-21800471)×1	3.17 Mb	Unknown	Pathogenicity Velocardiofacial/DiGeorge syndrome	Termination
5	Choroid plexus cysts	arr8p23.2p22 (2347605-17861146)×3	15.51 Mb	Unknown	Pathogenicity	Termination
6	Widened renal pelvis	arrXp21.1 (31713418-31825390)×0	112 Kb	Unknown	Pathogenicity Duchenne muscular dystrophy (DMD)/Becker muscular dystrophy (BMD)	Termination
7	Cardiac anomalies and right aortic arch	arr16p13.11 (14929071-16289059)×3	1.36 Mb	Inherited	Pathogenicity	No obvious abnormalities after delivery
8	Bilateral cleft lip and alveolar cleft	arr18q21.33q23 (60880283_78013728)x1	17.13 Mb	De novo	Pathogenicity SIMHA syndrome	Termination
9	Left widened lateral ventricle	arr 7q11.23 (72682113_74030629)x3	1.35 Mb	Unknown	Pathogenicity	Termination
10	Agenesis of corpus callosum	arr15q24.2 (75601120_75768740)x1	168 Kb	Inherited	Pathogenicity	Ear deformities. Hearing impairment. Delayed language development
11	Omphalocele	arr15q11.2 (22770422_23276605)x1	506 Kb	Unknown	Pathogenicity	Termination
12	Increased nuchal translucency thickening	arr16p11.2 (29428532_30190029)x1	761 Kb	Inherited	Pathogenicity	Termination
13	Right aortic arch with mirror, bilateral ventriculomegaly, polyhydramnios	arr17p11.2 (16727491_20463423)x1	3.74 Mb	Unknown	Pathogenicity	Termination
14	Multicystic dysplastic kidneys	arr15q11.2 (22770422_23276605)x1	506 Kb	Unknown	Pathogenicity	Termination
15	Increased nuchal translucency thickening	arr 16p13.11 (15338153_16327887)x3	990 Kb	Unknown	Pathogenicity	Termination
16	Growth retardation	arr15q11.2 (22770422_23288350)x1	518 Kb	Inherited	Pathogenicity	No obvious abnormal birth after 6 months

CMA, chromosomal microarray analysis; CNV, copy number variant; Mb, megabases; Kb, kilobases.

### Detection results and outcomes of 15 fetuses with VUS detected

There were 15 VUS samples, accounting for 4.76%. In 4 cases, the peripheral blood of the couple was collected for CMA detection. All four cases were inherited (Table 3).

**TABLE 3:** Detection results and outcomes of 15 fetuses with VUS detected.

Case	Ultrasound findings	CMA result	Fragment size	De novo/Inherited	Database query results	Follow-up outcome
1	Bilateral lateral ventricle broadening, widened posterior fossa pool	arrXq28 (151900478-152176976)×2	276 Kb	Unknown	Unclear clinical significance	No abnormalities in the newborn
2	Widened the left lateral ventricle	arr16p12.2 (21405328-21816543)×1	411 Kb	Unknown	Unclear clinical significance	No abnormalities in the newborn
3	Left diaphragmatic hernia and the heart was compressed to the right	arr2p23.3p23.2 (27484122-28582754)×3	1.10 Mb	Unknown	Unclear clinical significance	The pregnancy was terminated at 27 weeks due to fetal diaphragmatic hernia
4	Right side of the pelvic ectopic and a multicystic dysplastic kidney	arrXp11.23 (47859630-48252362)×3	393 Kb	Unknown	Unclear clinical significance	No abnormalities in the newborn
5	Double muscle under the tube membrane cyst, transparent insulation cavity broadening	arr12q21.32q21.33 (88609449_91720975)x1	3.11 Mb	Unknown	Unclear clinical significance	No abnormalities in the newborn
6	Ventricular septal defect	arr5q22.2q22.3 (112230357_113804572)x3	1.57 Mb	Unknown	Unclear clinical significance	No abnormalities in the newborn. After 6 months, the cardiac surgery should be consulted for ventricular septal defect surgery
7	Echogenic bowel	arr18q22.1 (63879972_66569034)x3	2.69 Mb	Inherited	Unclear clinical significance	No abnormalities in the newborn
8	Increased nuchal translucency thickening	arrXp22.33 (1358802_3520663)x3	2.16 Mb	Inherited	Unclear clinical significance	No abnormalities in the newborn
9	Fetal growth restriction (FGR)	arr 4q35.2 (187929332_188943890)x3	1.02 Mb	Inherited	Unclear clinical significance	The pregnancy was terminated at 32 weeks due to severe FGR
10	Ventricular septal defect	arr21q11.2q21.1 (15016487_17057220)x3	2.04 Mb	Unknown	Unclear clinical significance	Neonatal mild asphyxia and pneumonia. The rest showed no obvious abnormalities
11	Increased nuchal translucency thickening	arr16p13.3p13.2 (6896958_9417288)x3	2.52 Mb	Unknown	Unclear clinical significance	No abnormalities in the newborn
12	Atrial septal defect	arr 7p12.1 (51684538_53307764)x3	1.62 Mb	Unknown	Unclear clinical significance	No abnormalities in the newborn
13	Nasal bone calcification is not complete	arr12q21.31 (82919621_86264470)x3	3.34 Mb	Unknown	Unclear clinical significance	No abnormalities in the newborn
14	Aberrant left subclavian artery, widened bilateral lateral ventricle	arr7p15.2p14.3 (27858276_29464182)x3	1.61 Mb	Inherited	Unclear clinical significance	No abnormalities in the newborn
15	Increased nuchal translucency thickening	arr15q26.2q26.3 (97754343_98997760)x3	1.24 Mb	Unknown	Unclear clinical significance	No abnormalities in the newborn

CMA, chromosomal microarray analysis; CNV, copy number variant; Mb, megabases; Kb, kilobases.

## Discussion

Chromosomal karyotype analysis is the standard method for prenatal cytogenetic diagnosis and can detect aneuploidy, deletion/duplication of large fragments, and chromosomal structural recombination (Truong et al., 2023; Rosenblum et al., 2025). However, its resolution is low and it can only detect changes in genetic material with fragment sizes above 5–10 Mb (Oneda et al., 2017). CMA, also known as “molecular karyotype”, scans the genome at a genome-wide level and detects chromosomal imbalances in CNV, especially for the detection of imbalanced rearrangements such as microdeletion and microduplication (Kang et al., 2024). According to the chip design and detection principle, CMA technology can be divided into two categories: array-based comparativegenomic hybridization (aCGH) and single nucleotide polymorphism array (SNP array) (Lee et al., 2021). The chip that covers both CNV and SNP detection probes can have the characteristics of both CNV and SNP chips (Brady et al., 2012). In addition to CNVs, it can also detect most uniparental disomy (UPD) and triploidy, and can detect a certain level of mosaicism. In recent years, CMA has been more and more widely used in the field of prenatal diagnosis, and many studies have proved its advantages over fetal chromosomal karyotype analysis (Wang et al., 2022; Xue et al., 2024). CMA has the same efficiency as karyotyping for the detection of aneuploidy and unbalanced chromosomal rearrangements, and has higher resolution and sensitivity. CMA can also detect additional clinically significant genomic CNVs, especially for those with fetal structural abnormalities detected by prenatal ultrasound. CMA is one of the most effective genetic diagnosis methods (Hillman et al., 2013; Shaffer et al., 2012a; Shaffer et al., 2012b; Wapner et al., 2012).

In this study, 5.08% (16/315) P/LP CNVs were detected in addition to the normal karyotype detected by abnormal ultrasound. It is close to 6% reported by Wapner et al. (2012). Among them, the detection rate of P/LP CNVs in abnormal ultrasound soft markers was 4.42% (8/181). Akalın et al. reported that P/LP CNVs were detected in 6.5% fetuses with abnormalities in ultrasound soft markers (Akalın et al., 2023). The additional detection rate of abnormal chromosome by CMA in fetuses with abnormal ultrasound soft markers was 5.0% in the study of Jiang et al. (2024). Although the detection rate of abnormalities in ultrasound soft markers is slightly lower than that of structural abnormalities, given the large number of pregnant women in China, soft markers abnormalities are not rare and should not be easily ignored. Dynamic ultrasound follow-up is recommended. Invasive prenatal diagnosis and CMA can be recommended for those with no improvement on ultrasound review, or even aggravated or combined with multiple soft marker abnormalities. For CNVs identified by CMA, we classified P/LP CNVs by consulting authoritative databases and published literature, with a particular focus on known microdeletion/microduplication syndromes, in order to provide precise genetic evidence for clinical genetic counseling and to prevent the birth of fetuses with severe genetic disorders.

Some microdeletions/microduplications associated with neurodevelopmental disease loci are sometimes detected. They result in a highly variable, pathogenic, and low penetrance clinical phenotype, largely inherited from parents with no or mild phenotypes. For example, 15q11.2 (BP1-BP2) microdeletion CNVs were detected in cases 11, 14, and 16 of Table 2. Some scholars believed that such CNVs with mild clinical manifestations and low penetrance should be considered as unreported to clinicians and couples (Jønch et al., 2019; Maya et al., 2020). However, in accordance with current clinical practice and genetic counseling guidelines in China, we chose to report these low penetrance, susceptibility-related CNVs to genetic counselors. It ensures that couples receive comprehensive information regarding potential clinical implications, thereby allowing for fully informed decision-making during prenatal genetic counseling. After full counseling and parental verification of the results, case 11 chose to continue the pregnancy, and no abnormalities were found in the follow-up after birth. Pregnancy was terminated in case 14 and case 16 due to other structural abnormalities. The detection rate of VUS was 4.76% in our study, which was close to 3.4% in the literature (Wapner et al., 2012). In many cases, the interpretation of VUS is the main difficulty. Some of these cases are rare *de novo* mutations, and some are related to the penetrance of the mutated gene, which means that the fetus is predisposed to a genetic disease but does not always develop it, such as autism. Parental sample detection and pedigree analysis are helpful for the interpretation of VUS results. However, based on the current understanding of the human genome and the accumulation of relevant databases, it is still impossible to accurately characterize all genomic information and its correlation with diseases. This situation often leads to anxiety for pregnant women and their families and even to wrongful termination of pregnancy.

In addition, two fetuses were found to have abnormalities after birth when CMA was normal. This may be due to the fact that chromosomal microarray analysis (CMA) is designed to detect copy number variations (CNVs) but cannot identify single-nucleotide variants (SNVs) or small insertions/deletions (InDels) at the sequence level (Similuk et al., 2022). Therefore, late prenatal examination or combined with other techniques is the key to avoid birth defects. It has been reported that whole exome sequencing (WES) can diagnose 25%–35% of unexplained genetic diseases in patients with negative results of karyotype analysis and CMA (2015). Another study has shown that in cases where karyotype and CMA assessment of fetal malformations cannot identify chromosomal abnormalities, whole exome sequencing will help in the diagnosis of fetal structural abnormalities, increasing the diagnostic rate by about 10% (Petrovski et al., 2019). In this study, pregnant women’s compliance with WES testing was poor, the number of fetuses undergoing WES testing was small, and the obtained test data was insufficient for statistical analysis. WES has certain application value in prenatal diagnosis, but its clinical application and popularization are confronted with numerous challenges.

Furthermore, this study found that the positive detection rate of CMA in fetuses with isolated ultrasound soft marker abnormalities was lower than that in fetuses with structural ultrasound abnormalities, which was consistent with the conclusions of multiple previous studies (Kim et al., 2024; Jiang et al., 2024; Song et al., 2024). Isolated ultrasound soft marker abnormalities are mostly transient manifestations during fetal development, and their correlation with chromosomal abnormalities remains to be further elucidated (Moradi et al., 2024). Over-reliance on CMA testing may lead to unnecessary increases in medical costs and heavier psychological burdens on pregnant women. Therefore, in clinical practice, the application of CMA for such fetuses should be comprehensively evaluated in combination with multiple indicators such as maternal age and serological screening results. Meanwhile, the limitations and uncertainties of the test should be fully informed to the family members to avoid over-interpretation of the test results. In addition, CMA is limited in its ability to detect low-level chromosomal mosaicism. In general, conventional CMA platforms may not reliably identify mosaicism below a 20%–30% cellular level (Jiazhen et al., 2025). Low-level mosaicism, especially those below this detection threshold, cannot be completely excluded even with a normal CMA result (Deng et al., 2025). Therefore, in cases with a high clinical suspicion of mosaicism but normal CMA findings, complementary methods such as chromosomal microarray with enhanced sensitivity, QF-PCR, or karyotyping after extended culture may be considered for further verification (Jiazhen et al., 2025). This limitation should be taken into consideration during prenatal genetic counseling.

This study confirmed through CMA of 315 fetuses with ultrasound abnormalities that this technique can improve the detection rate of chromosomal abnormalities in such fetuses, thereby providing a crucial molecular basis for fetal prognosis evaluation and clinical genetic counseling. However, this study has certain limitations. First, it adopted a single-center retrospective study design. The included subjects were pregnant women who had fetal ultrasonic abnormalities and voluntarily underwent invasive prenatal diagnosis in our hospital, which inevitably led to selection bias. The homogeneity of the study population restricted the extrapolation of the research results, and high-risk pregnant women with normal ultrasonic findings were not included as controls for comparison. Second, the diagnostic efficacy of CMA was not analyzed in a stratified manner based on ultrasonic structural anomalies across different systems, which makes it difficult to provide targeted recommendations for prenatal diagnostic strategies. Third, due to the limited sample size, this study failed to summarize the variant regions with distinct characteristics corresponding to different types of fetal ultrasonic abnormalities. Finally, this study has certain limitations in the follow-up phase. Constrained by the relatively short follow-up duration, it failed to comprehensively collect information on the long-term postnatal developmental outcomes and clinical prognosis of the fetuses, thus hindering further verification of the long-term application value of CMA in prognostic assessment. Therefore, larger-sample-size studies are required to enrich relevant data. Future research should be improved through multicenter prospective studies, integration of multi-omics technologies, and improvement of follow-up databases.

## Conclusion

CMA technology can detect and precisely locate chromosomal copy number variants in the whole genome, with the advantages of high resolution, throughput and accuracy, and does not require cell culture. Compared with traditional chromosomal karyotype analysis, it has obvious advantages, and the technology platform is relatively mature. CMA can improve the detection rate of chromosomal abnormalities in fetuses with ultrasound abnormalities, thereby providing a crucial molecular basis for fetal prognostic assessment and clinical genetic counseling. It is a supplement to the traditional chromosomal karyotype analysis, which has improved the efficiency of prenatal diagnosis, and reduced the incidence of fetal birth defects.

## Data Availability

The original contributions presented in the study are included in the article/supplementary material, further inquiries can be directed to the corresponding author.
